# Treatment of Septic Peritonitis Consequent upon Miscarriage and Pyosalpinx

**Published:** 1902-05

**Authors:** 


### TREATMENT OF SEPTIC PERITONITIS CONSEQUENT UPON MISCARRIAGE AND PYOSALPINX.

—— B., age 21; brought to hospital in ambulance, January 24. Three weeks prior to admission had a miscarriage—received no medical attention; period of utero-gestation was four months. Has had gonorrhea. Temperature on admission was 104°. Patient complained of severe pain in pelvic region.

Examination revealed a profuse vaginal discharge, tenderness and tympani in the pelvic region. There was a large mass in right side of pelvis, tenderness and all evidences of peritonitis. Patient was prepared for curetment. Uterine cavity was thoroughly cleansed, a dull curet being used, after which it was drained. Uterus was not tamponed.

February 3, patient was prepared for celiotomy, as symptoms did not materially improve. The bowels, adnexa and omentum were bound together by adhesions. There was a large pyosalpinx on right side. This was removed after adhesions had been broken up. The uterus was enlarged to about twice its size and there was a great amount of oozing from the inflamed surface. Abdominal cavity was flushed with normal salt solution, the fluid left in the abdominal cavity and wound closed. Temperature dropped to normal two days after operation and remained so until the 21st day, when patient was discharged.

